# Importin α3 Is Tolerant to Nuclear Localization Signal Chirality

**DOI:** 10.3390/ijms26167818

**Published:** 2025-08-13

**Authors:** Felipe Hornos, Bruno Rizzuti, José L. Neira

**Affiliations:** 1 IDIBE, Instituto de investigación, Desarrollo e Innovación en Biotecnologia Sanitaria de Elche, Universidad Miguel Hernández, 03202 Elche, Alicante, Spain; fhornos@umh.es; 2 CNR-NANOTEC, Institute of Nanotechnology of Consiglio Nazionale delle Ricerche, SS Rende (CS), Department of Physics, University of Calabria, 87036 Rende, Italy; 3 Instituto de Biocomputación y Física de Sistemas Complejos, Universidad de Zaragoza, 50018 Zaragoza, Spain

**Keywords:** nuclear localization signal, D-enantiomers, disordered peptides, binding, calorimetry, fluorescence, molecular simulations

## Abstract

Several carrier proteins are involved in nuclear translocation from the cytoplasm to the nucleus in eukaryotic cells. We have previously demonstrated the binding of several intact folded and disordered proteins to the human isoform importin α3 (Impα3); furthermore, disordered peptides, corresponding to their nuclear localization signals (NLSs), also interact with Impα3. These proteins and their isolated NLSs also bind to the truncated importin species ∆Impα3, which does not contain the N-terminal disordered importin binding domain (IBB). In this work, we added a further ‘layer’ of conformational disorder to our studies, testing whether the isolated D-enantiomers of NLSs of selected proteins, either folded or unfolded, were capable of binding to both Impα3 and ∆Impα3. The D-enantiomers, like their L-form counterparts, were monomeric and disordered in isolation, as shown by nuclear magnetic resonance (NMR). We measured the ability of such D-enantiomeric NLSs to interact with both importin species by using fluorescence, biolayer interferometry (BLI), isothermal titration calorimetry (ITC), and molecular simulations. In all cases, the binding affinities were within the same range as those measured for their L-isomer counterparts for either Impα3 or ∆Impα3, and the binding locations corresponded to the major NLS binding site of the protein. Thus, the stereoisomeric nature is not important in defining the binding of proteins to the main component of classical cellular translocation machinery, although the primary structure of the hot-spot site for NLS binding of importin is well defined.

## 1. Introduction

Some proteins carry out their function within the nucleus, after being synthesized in the cytoplasm; then, such proteins must be translocated through the nuclear pore complex (NPC). Nuclear translocation generally occurs through importins, together with other auxiliary proteins [1,2]. The classical, well-known nuclear import pathway is triggered by the recognition of a nuclear localization signal (NLS) region in the corresponding cargo protein by importin α [1]. The cargo–importin α complex then binds to importin β, and the ternary complex moves through the NPC. Importin α is any member of a family of highly similar proteins with a distinct modular structure, which includes several α-helix repeat armadillo (ARM) units [1,2]. It is composed of two domains: (i) a 60-residue-long, disordered, N-terminal importin β-binding domain (IBB), which is used for binding to importin β before transport through the NPC; and (ii) a larger, well-folded, C-terminal domain that included the NLS binding region and is formed by ten ARM units [3]. When importin β is not present, the IBB domain, which mimics an NLS polypeptide, occupies the ARM regions implicated in NLS recognition [3]. This intramolecular interaction has an auto-inhibitory role, and it is also relevant in cargo dissociation on the nucleoplasm side [3].

In the past, we have studied the binding of several proteins to Impα3 (also called KPNA4), which is one of the human isoforms of importin α, and to its truncated species, ∆Impα3, in which the IBB has been removed [4,5,6]. We considered Impα3 as a model of nuclear translocation for several proteins because of its greater flexibility compared with other importins; this feature confers Impα3 a greater ability to interact with various protein cargos [4]. In addition, from an experimental point of view, Impα3 can also be easily expressed and purified for in vitro studies [4,5,6]. We studied the binding of both importin species, Impα3 and ∆Impα3, to (i) the wild-type isolated NLS region of NUPR1, a disordered protein, comprising residues 54 to 74; and (ii) a variant of this NLS region with a post-translational modification, with the key residue Thr68 phosphorylated [6]. Similarly, the binding to both importin species of the disordered NUPR1 paralogue, NUPR1L, and its isolated NLS region, NLS-NUPR1L, has been described [5]. We also studied the binding of both importin species to the well-folded enzyme PADI4 and its isolated NLS [7]. In all cases, the binding affinities of each importin species to either the corresponding intact protein or to the isolated NLS were similar. We believe that, by identifying the driving interactions that govern the binding of several NLSs to a model importin, we shall be able to design new drugs or small peptides that hamper such binding, selectively inhibiting nuclear translocation at the cellular level to obtain a therapeutic effect [8]. These compounds, capable of binding to importins, will hamper the binding of protein cargos, which can function in the nucleus as transcription factors, as enzymes modifying other nuclear proteins (probably histones), or proteins intervening in cell cycling. Alternatively, those cargos can be also involved in protein networks and in DNA binding and its regulation. In this way, upon addition of the compound to the medium the cargo protein will be inhibited from carrying out its function within the nucleus, and, therefore, the assayed compound can prevent the development of some malignancies, including cancer and viral infections [9,10,11,12,13,14].

Proteins, like many other biomolecules, are composed of chiral building blocks. Ribosomes recruit L-chiral amino acid, and recombinant proteins are constructed within an L-framework. Although they are far less common in biological contexts and more difficult to prepare by biosynthetic routes, the mirror-image D-amino acids, which constitute proteins that fold into the opposite chirality, have remarkable potential in scientific research, and they are used throughout nature with different functions (see, for instance, [15]). The D-enantiomeric proteins can be used for (i) the creation of mirror-image life; (ii) mechanistic studies of natural proteins; and (iii) the design and isolation of ultra-stable binders that are both non-proteolytic—i.e., non-recognizable by endogenous proteases—and non-immunogenic [16]. Currently, the design of drugs that target proteins can take two approaches: (i) “small-molecule” drugs (usually with a molecular weight lower than 500 Da), and (ii) protein/peptide-based drugs (usually with a molecular weight larger than 1000 Da) [17]. Small molecules have a favorable oral availability and are more commonly obtained by a rational design [18], but sometimes they may show low target specificity, leading to unwanted side effects. On the other hand, polypeptides bind to target proteins with high affinity and specificity, and they can be obtained synthetically by well-established and cost-efficient methods [19]. The main disadvantages of peptide-based drugs are their potential immunogenicity and low bioavailability due to degradation and short half-lives. These shortcomings can be overcome by the use of D-amino acids. In this context, we wondered whether the same affinities for Impα3 and ∆Impα3 could be observed for the D-enantiomers of the isolated NLS regions, leading to the possibility of using these D-NLS peptides (or another NLS-mimicking peptide) as peptide-based drugs against Impα3.

To answer these questions, we started from peptides completely made of D-enantiomers of the corresponding NLS regions (hereafter, for brevity, named D-NLSs) of the above-mentioned proteins. We studied the binding of such polypeptide regions to Impα3 and ∆Impα3, by using fluorescence, BLI, ITC and molecular docking. The use of Impα3 as a model protein to monitor the binding of stereoisomers of the NLSs could be biased due to the high flexibility of this importin isoform; however, since there are no other related studies, our findings could provide a first insight towards the behavior of the translation machinery towards the D- and L-enantiomers. In this work, we also explored the conformational preferences of the D-enantiomers in comparison with their natural L-form counterparts, by using NMR. The D-NLSs were observed to be, in all cases, monomeric and disordered in solution, as it happens with the corresponding natural L-NLSs. Furthermore, the D-enantiomeric peptides were capable of binding to either Impα3 or ∆Impα3 with a similar binding affinity as their L-form counterparts (as concluded from ITC and BLI assays) and in the same binding location, as indicated by molecular dynamics. Taken together, our in vitro and in silico results suggest that enantiomeric features are not critical in defining nuclear translocation, and, therefore, D-peptides, with better non-immunogenic properties and a higher stability than natural L-peptides, could be conveniently used to target importins.

## 2. Results

### 2.1. The D-NLS Peptides Were Monomeric and Disordered in Aqueous Solution

**Figure 1:**
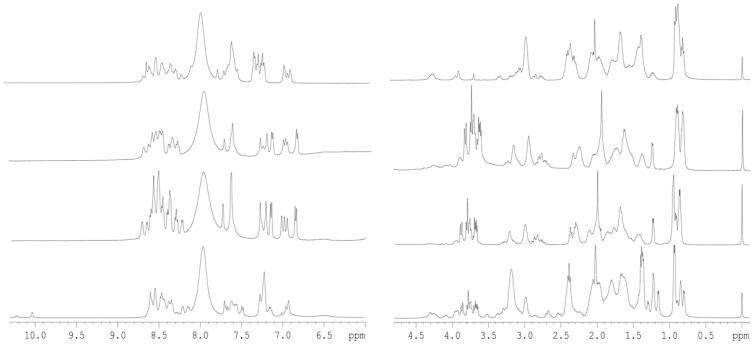
1D ^1^H-NMR spectra of D-NLS peptides: The amide (**left**) and aliphatic (**right**) regions of the 1D ^1^H-NMR spectra of the peptides are shown. From the bottom to the top, the D-NLS peptides are: D-NLS-NUPR1L, D-NLS-NUPR1, D-NLS-NUPR1phospho and D-NLS-PADI. All spectra were acquired at 10 °C. The signal on the right side of the methyl regions corresponds to the TSP.

The conformational features of the four isolated D-NLS peptides were explored by using the 1D-^1^H-NMR spectrum (Figure 1). The spectra of each peptide showed, in all cases, a clustering of the signals of the amide protons between 8.0 and 8.7 ppm, whereas the methyl protons were observed grouped between 0.8 and 1.0 ppm. All these values are typical of mostly disordered chains [20,21,22].

The diffusion coefficient, *D*, values measured in the DOSY experiment are reported in Table 1. For the four peptides, we measured the experimental value of *D* for dioxane ranging from (1.2 ± 0.1) × 10^−5^ to (1.05 ± 0.02) × 10^−5^ cm^2^ s^−1^ in each solution containing the corresponding peptide; we assumed a hydrodynamic radius of 2.12 Å for dioxane [23] (Table 1) under any condition. The estimated values of *R*_h_ for the D-NLS peptides were also comparable to those obtained theoretically [24] for a monomeric random-coil polypeptide with the same molecular weight as the corresponding D-peptide. Furthermore, the values of *D* were similar for the D- and L-peptides, and the latter were already being described as disordered [5,6,7]. Thus, we can conclude that the four D-peptides were monomeric and disordered, like the L-form counterparts, and, then, there was no difference in the hydrodynamic properties of the peptides by changing the enantiomeric nature of the residues.

**Figure 2:**
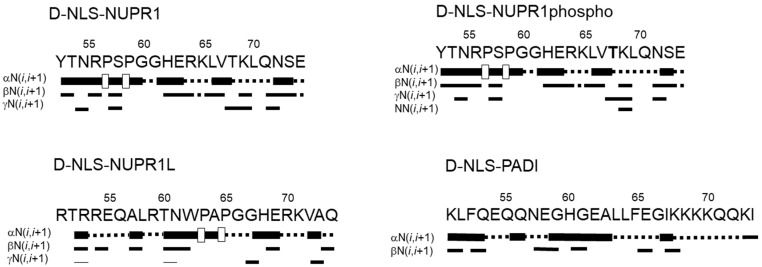
NOE diagrams of the D-NLS peptide series in aqueous solution: NOEs are classified as strong, medium or weak, and represented by the height of the bar underneath the sequence; signal intensity was judged from the NOESY experiments. The dotted lines indicate NOE contacts that could not be unambiguously assigned. A white square indicate αδ*(i*,*i* + 1) NOEs involving a proline residue. The post-translational modified reside Thr68 appears in bold in the peptide derived from NUPR1.

To further confirm the mainly disordered nature of the D-NLS peptides, we carried out homonuclear 2D-^1^H-NMR experiments (Tables S1–S4). Although we were able to obtain, for all the peptides, good TOCSY and NOESY spectra to allow for resonance assignments, for some of the peptides, we could not fully assign them due to the large number of glutamine, glutamic acid, and lysine residues. The peptides were mainly disordered in solution, as suggested by two types of evidence described in the next lines, further confirming the results from DOSY experiments (Table 1) and 1D-^1^H-NMR spectra (Figure 1). The first piece of evidence is based on the conformational shifts: the sequence-corrected conformational shifts (∆δ) of H_α_ protons [20,21,22] for unambiguously assigned residues were within the commonly accepted range for random-coil peptides (∆δ ≤ 0.1 ppm) (Tables S1–S4). The values of the chemical shifts for the protons of all residues were similar to those measured for the L-form counterparts, and, therefore, the different stereoisomeric features did not alter the chemical shifts, as has been observed in other examples when the D- and L-forms of peptides have been assigned [25]. And the second piece of evidence is based on the type of NOEs observed: no long- or medium-range NOEs were detected but only sequential ones (namely, αN (*i*, *i* + 1), αδ(*i*, *i* + 1) (the latter for the proline residues) and βN (*i*, *i* + 1)) in the polypeptide patches fully assigned (Figure 2). The pattern of observed NOEs in each D-NLS peptide was the same as observed for the corresponding L-peptide counterparts [5,6,7].

### 2.2. The D-NLS Peptides Bound to Impα3 or ∆Impα3 with a Low Affinity

To test whether the D-NLSs interacted with Impα3 and ∆Impα3 in vitro, we followed a three-part experimental approach, by using fluorescence, BLI and ITC to quantitatively measure the thermodynamic parameters of the possible binding.

**Figure 3:**
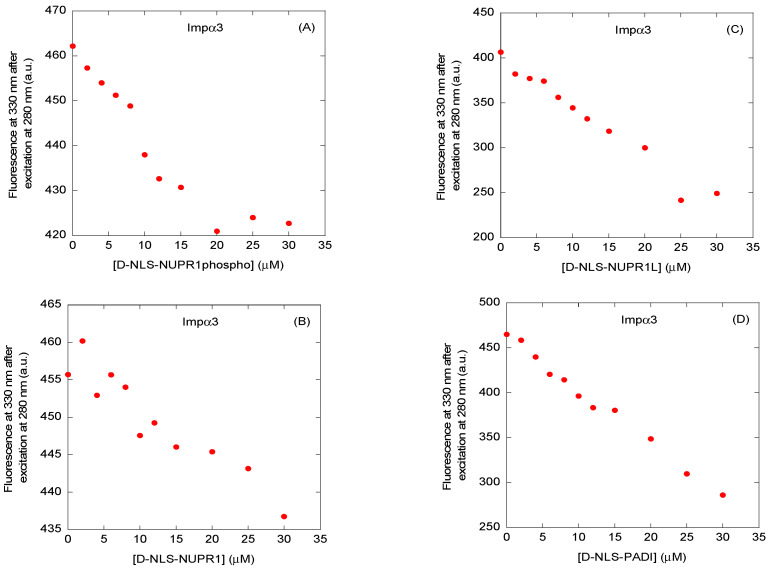
Binding to Impα3 of the D-NLS peptides, as monitored by fluorescence: fluorescence titrations of Impα3 with (**A**) D-NLS-NUPR1phospho; (**B**) D-NLS-NUPR1; (**C**) D-NLS-NUPR1L; and (**D**) D-NLS-PADI. The fluorescence intensity on the y-axis is the relative fluorescence intensity after removal of the corresponding blank. Experiments were carried out at 25 °C.

We first carried out fluorescence titrations with each of the four peptides and either Impα3 or ∆Impα3, by increasing the concentration of the corresponding peptide. The results indicate that, for both importin species with any of the four peptides, there was a decrease in the fluorescence intensity, after either excitation at 280 or 295 nm, and correction for inner-filter effects [26], thus suggesting the occurrence of binding (Figure 3 and Figure S1 and Table 2 and Table 3). However, under the conditions explored, we were not able to determine a reliable *K*_d_ value for most of the titration curves of the peptides (either after excitation at 280 or 295 nm). The impossibility of obtaining trustworthy titration curves was due probably to the fact that such thermodynamic parameters were beyond the concentration range accessible using this technique (i.e., possibly having higher or lower *K*_d_ values than the explored peptide concentration), at least at the values we used for the D-peptides and/or importin species [27,28]. Although we could not determine consistent *K*_d_ values, our results suggest that, since most of the peptides contained tyrosine or phenylalanine residues (Table 1), the binding involved at least some of the tryptophans of the importin species (both Impα3 and ∆Impα3 have six tryptophans). Only for D-NLS-NUPR1L, whose sequence contained a tryptophan residue (Table 1), we could not ascertain the involvement of the indole side chains of Impα3 and ∆Impα3 in its binding.

**Figure 4:**
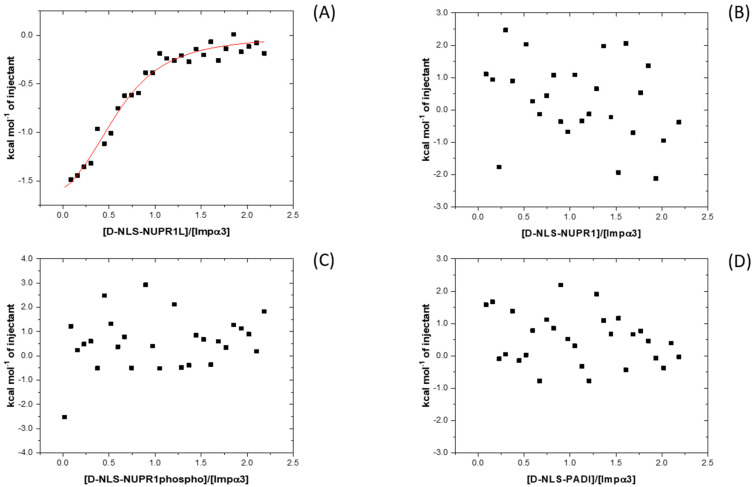
Binding to Impα3 of the D-NLS peptides as monitored by ITC: calorimetric titrations of Impα3 with (**A**) D-NLS-NUPR1L; (**B**) D-NLS-NUPR1; (**C**) D-NLS-NUPR1phospho; and (**D**) D-NLS-PADI. Continuous line corresponds to the fitting curve according to a single ligand binding site interaction model. Experiments were carried out at 25 °C.

We also used ITC to determine the thermodynamic binding parameters in the association of each of the peptides to both importin species (Figure 4). The results indicated that, except for D-NLS-NUPR1L (for which *K*_d_ values close to ~ 5 μM for both importin species were measured, Table 2 and Table 3), the interaction of each importin species with any of the D-NLS peptides did not yield any binding isotherm (Figure S2). For D-NLS-NUPR1L, the values of enthalpy, ∆*H*, were −2.5 ± 0.2 and −4.5 ± 0.7 kcal mol^−1^ for the binding to Impα3 and ∆Impα3, respectively. On the other hand, the values previously obtained for the L-form counterpart were −3.1 ± 0.5 and −2.4 ± 0.5 kcal mol^−1^ for Impα3 and ∆Impα3, respectively [5], which are similar to those observed for D-NLS-NUPR1L. These results suggest that, since there was binding between all D-NLSs and the two importins (as suggested by the fluorescence titrations), the heat exchanged upon binding of the other D-NLS peptides was small enough to be measured by ITC under our conditions. Then, although we could not measure the ITC thermograms for the rest of the peptides, the findings that, (i) for the D-NLS-NUPR1L, the ITC thermograms yielded similar parameters of binding as for the L-form counterpart [5], and (ii) the apparent *K*_d_ values obtained from BLI measurements (see next paragraph) for the rest of the D-isomers were similar to the *K*_d_ values obtained by ITC for the corresponding L-form counterparts [5,6,7] suggested that the affinities for either Impα3 and ∆Impα3 were similar for both stereoisomers.

**Figure 5:**
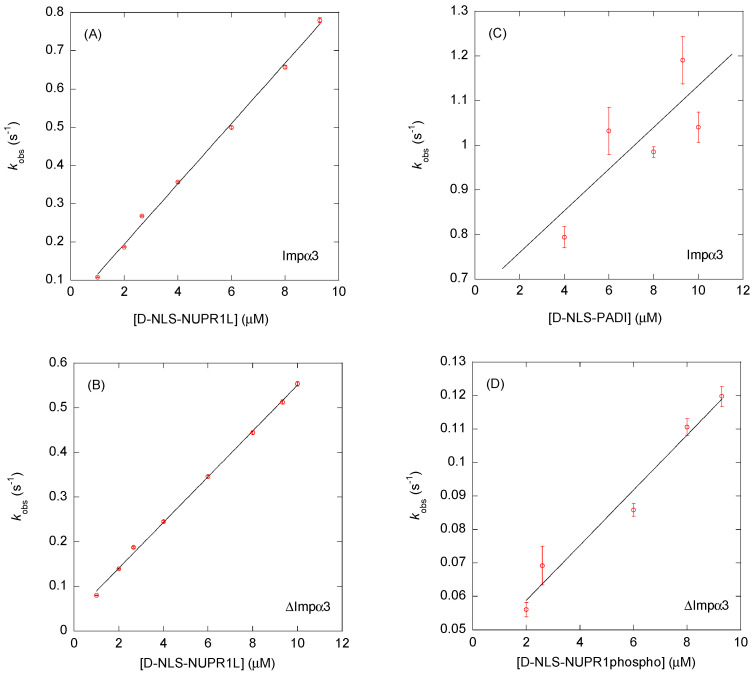
Binding to Impα3 and ∆Impα3 of selected D-NLS peptides as monitored by BLI: pseudo-first-order plots (Equation (5)) of the binding of: (**A**) D-NLS-NUPR1L to Impα3; (**B**) D-NLS-NUPR1L to ∆Impα3; (**C**) D-NLS-PADI to Impα3; and (**D**) D-NLS-NUPR1phospho to ∆Impα3. The error bars for each of the peptide concentrations in the four panels are fitting errors to the exponentials of the sensorgrams (Equation (4)). Experiments were carried out at 25 °C.

Then, as a further way to quantitatively study the binding process, we used BLI. With the help of this technique, we were able to determine an apparent affinity constant for each peptide, except for the wild-type D-NLS-NUPR1, where the pseudo-first-order plot led to a negative y-axis intercept (Figure 5, Table 2 and Table 3). It is important to stress that the apparent *K*_d_ (= *k*_off_/*k*_on_) values determined by using BLI data were obtained under the assumption that the binding of the peptide to each of the importin species could be modeled as a two-state process. Under this assumption, it could happen that some of the *K*_d_ values determined by BLI did not agree with those determined by fluorescence or ITC. For all the peptides, we observed reliable sensorgrams (Figure 5 and Figure S3), thus confirming the fluorescence results: there was an association between each of the importin species with the corresponding peptide. The association rates, *k*_on_, for the same peptide with both importin species were similar in most cases, except for D-NLS-NUPR1phospho (Table 2 and Table 3). The smaller value of the *k*_on_ rate of D-NLS-NUPR1phospho for ∆Impα3, indicating a slower association process compared to the same rate in the binding to Impα3, might be due to electrostatic effects, which are different in the phosphorylated species compared to the wild-type peptide.

It is interesting to compare, at least for one available example, the values of the association (*k*_on_) and dissociation (*k*_off_) rates for both stereoisomers in the binding to both importin species. We previously reported the binding of L-NLS-PADI to Impα3 and ∆Impα3 [5]. The values of the kinetic rates were as follows: *k*_on_ = 0.003 ± 0.002 μM^−1^ s^−1^ and *k*_off_ = 0.32 ± 0.01 s^−1^ (for Impα3); and *k*_on_ = 0.020 ± 0.003 μM^−1^ s^−1^ and *k*_off_ = 0.23 ± 0.01 s^−1^ (∆Impα3). Then, the values of the *k*_off_ rates for each of the importin species were similar for both stereoisomers (Table 2 and Table 3), but the values of the *k*_on_ rates for each of the importin species were smaller (and, therefore, slower) for the L-form counterparts. Further experiments with other D- and L-peptides, and not only involving the translocation machinery, will be necessary to confirm these findings.

From the measured values of *k*_on_ and *k*_off_, and the determined apparent *K*_d_ ones, for the four peptides, we can stress several conclusions.

First, in general terms, we could observe that the apparent *K*_d_ values of the D-peptides were always larger for Impα3 than for ∆Impα3, in agreement to what happens with the L-form counterparts, in the cases where a measurement was possible [7]. This is due to the removal of the 60-residue-long IBB n in the truncated protein species, which competes for the binding to the NLS recognition site present in importin, tending to hamper the association of the protein cargo through the NLS regions. Then, the D-NLS peptides followed the same trend, by using BLI, as the L-form counterparts in binding to each importin species.

Second, we could observe that the values of the *k*_on_ and *k*_off_ rates, as well as the affinity constant ones, varied among the peptides. Then, the binding reaction was modulated by both the nature of the importin species (with or without the IBB) and each particular type of the NLS region. Similar conclusions were obtained for the L-enantiomeric NLSs [5,6,7].

Next, we observed that for D-NLS-NUPR1L, which had the smallest apparent *K*_d_ constant from BLI experiments (~1 μM) (Table 2 and Table 3), we were able to measure ITC binding isotherms for both importin species (Figure 4 and Figure S2A). The rest of the peptides yielded apparent *K*_d_ constants, as determined by BLI, in the range of 10–15 μM. The small affinity determined by BLI for D-NLS-NUPR1L was beyond the range of concentrations explored by fluorescence for both importin species [27,28]. Therefore, these *K*_d_ values, determined by BLI, could explain the lack of reliable results in determining such a constant by fluorescence (Figure 3 and Figure S2). The same explanation holds in light of the large apparent *K*_d_ values of D-NLS-NUPR1phospho and D-NLS-PADI (Table 2 and Table 3) for both importin species. In addition, it is interesting to pinpoint that the binding of D-NLS-NUPR1phospho, when compared to that of the wild-type peptide, D-NLS-NUPR1, appeared modified to the same extent as its L-form differed from its wild-type L-counterpart [6].

Finally, as stated above, it is interesting to note that the *K*_d_ values from BLI experiments, when compared to those measured by fluorescence and/or ITC for their L-form counterparts [5,6,7], were similar (Table 2 and Table 3). These results indicate that the D-peptides can bind to either Impα3 or ∆Impα3 with similar affinities as those of the L-peptides.

### 2.3. The D-NLS Peptides Targeted the Major NLS Binding Site of Importin α3

The binding of the D-NLS peptides on the surface of ∆Impα3 was investigated via molecular docking simulations. The use of such a computational technique was motivated by the difficulties in using more traditional experiments (e.g., crystallography) to identify the binding location, due to the intrinsically disordered nature of these peptides evidenced by our NMR measurements (Figure 1 and Figure 2, Tables S1–S4). For the same reason, more complex simulation techniques (e.g., classical molecular dynamics) could not be used to model the binding due to the large conformational flexibility expected for the peptides [29]. Our docking simulations were performed at the atomic level, with the sole exception of implicit apolar hydrogens, and by considering possible rotations about each peptide dihedral angle. Furthermore, the calculations were carried out in a completely blind fashion, considering a search volume encompassing the entire protein surface and with no prior bias towards any particular binding location. Finally, a high exhaustiveness was used in the search procedure [30].

**Figure 6:**
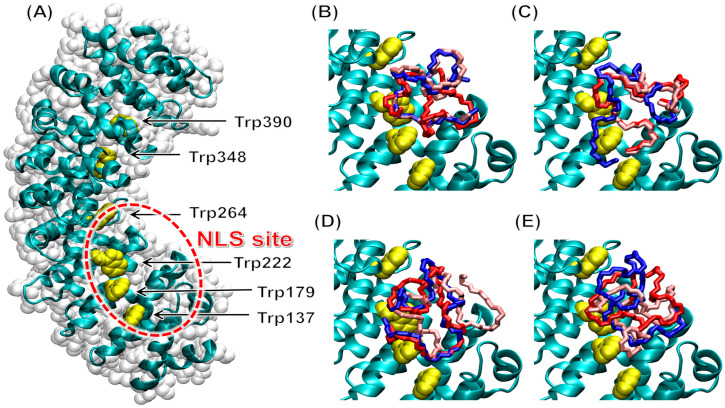
Binding to ∆Impα3 of the D-NLS peptides in molecular docking simulation: (**A**) structure of free ∆Impα3, organized in α-helix repeat ARM units, with the N terminus at the bottom and C terminus on the top; the tryptophan residues (yellow) and the major NLS binding site (red circle) are labeled. Docking poses of the D-NLS peptides are shown for (**B**) D-NLS-NUPR1; (**C**) D-NLS- NUPR1phospho; (**D**) D-NLS-NUPR1L; and (**E**) D-NLS-PADI. For clarity, the three most favorable docking poses are reported (1st: red; 2nd: blue; 3rd: pink), and the sole backbone (N–C α–C) of the peptides is shown.

The results (Figure 6) reveal that the most favorable docking poses obtained for each of our D-NLS peptides were all found to target a well-defined hot spot within the innermost surface of ∆Impα3, approximately centered on residues Trp179 and Trp222; this region corresponds to the major NLS binding site of the protein. This result agrees with the findings obtained in our fluorescence titrations (Figure 3), indicating that some tryptophans of Impα3 were involved in the binding (the number of tryptophan and tyrosine residues in both importin species is the same). The different binding modes observed in simulations showed some conformational variations, which were consistent with the disordered nature expected for these peptides, but might also be due, at least in part, to the difficulties of obtaining exhaustive sampling due to the large number of degrees of freedom of the peptides. A moderate affinity of different portions of the NLS region towards the same target site could also favor the first steps of the binding recognition process and hamper its dissociation when bound [7,31], or, alternatively, help in the context of an induced fit binding mechanism.

The binding energies calculated corresponded to moderate affinities, up to −5.3 kcal mol^−1^ (Table 4), roughly approaching a binding equilibrium in the intermediate micromolar range. These results are essentially in agreement with our experimental observations (Table 2 and Table 3), but they might also be affected by relatively large uncertainties due to the possibility of incomplete sampling, as already mentioned above. They might also include enthalpy/entropy compensation effects, due to a tendency to underestimate the entropic contribution to the binding energies and overestimate the enthalpic one, for such flexible ligands. Therefore, the affinities calculated in the experiments in vitro could be considered more accurate than the ones obtained in simulation.

Our results were also in good agreement, both in terms of anchoring location and binding affinity, with those we have observed for several L-NLS peptides, both paralleling the D-NLS regions studied here [5,6,7] or corresponding to the NLS of other proteins [31,32]. Therefore, two strong conclusions based on our simulation findings are as follows: (i) the stereochemistry of the peptides was not essential to drive the association to Impα3; and (ii) both the D-NLS and L-NLS enantiomeric species of our peptides targeted the major NLS binding site.

## 3. Discussion

Mirror-image peptides, composed of D-amino acids, are an attractive therapeutic option, as they exhibit high metabolic stability and lack of immunogenicity compared to their L-form counterparts. The development of mirror-image binding peptides is achieved through chemical synthesis of D-target residues and the selection of L-binders from a phage display library; then, D-peptides possibly capable of binding to the physiological L-targets can be synthesized [33,34]. Recently, it has also been shown that designed and synthesized mirror-image polypeptides can bind directly to their natural L-target [33].

In this work, we were interested in elucidating whether mirror-image D-peptides, which comprise the NLS regions of different proteins, were also capable of binding to Impα3 and its truncated species, without the IBB, ∆Impα3. This (L-chirality) target is important because Impα3 shows high conservation across different species [31,35], provides nuclear translocation to a large variety of cargo polypeptides [4], and constitutes an attractive pharmaceutical target to inhibit or regulate the traffic of proteins towards the cell nucleus. For these reasons, we previously investigated, in detail, the binding of NLS peptides in L-form to importin α3 [5,6,7,31,32]. In fact, we have previously shown that, for all the NLS peptides explored in this work, binding of their corresponding L-form occurred at the same location both for Impα3 and ∆Impα3, and in the latter, it was facilitated (i.e., larger affinity) by the lack of the IBB. Furthermore, the L-NLS peptides remained disordered in the corresponding complex with intact importin, as shown by docking and molecular dynamics simulations [5,6,7,31,32], with the exception of a short recognition motif transiently anchoring to the major NLS binding site.

By extending our study to D-peptides, from the combination of all of our experimental and computational findings on the D-NLS peptides here reported, we can pinpoint three main results. First, chirality did not seem to affect the disordered nature of the isolated NLS region (Table 1). Second, the binding location of both the D-NLS and L-NLS peptides was the same and corresponded to the major NLS binding site of Impα3, as indicated by our molecular simulations (Figure 6). And, third, chirality did not dramatically alter the affinity parameters of the association reaction (Table 2 and Table 3), yielding values of the binding affinity within the same range of those measured for the L-form counterparts [5,6,7]. Studies on other D-peptides, derived from the viral macrophage inflammatory protein-II, have shown that the affinity for the corresponding L-protein, a chemokine receptor, was higher than that of the corresponding L-peptide counterpart [36], but, in this case, both the D- and L-peptides had a small population of turn-like structures around a histidine residue. Recent results on the importance of stereochemistry in the binding of disordered proteins have suggested that chirality is not important when the complex is fully disordered; only when the ligand becomes ordered upon association (i.e., in the presence of a folding-upon-binding process), a proper stereochemistry is critical to attain binding [25]. In the case of the D-NLS peptides studied in this work, there was binding (as shown by the three biophysical probes used and the docking technique, Table 2, Table 3 and Table 4), and, since all the NLS regions remained disordered, the affinities were not substantially altered upon changing the peptide stereochemistry.

A few additional conclusions can be drawn from our results, in the context of their relevance for understanding the importance of importin in the regulation of the nucleocytoplasmic transport of cargo proteins. Although we cannot rule out that the intrinsic high flexibility of Impα3 could be involved in its lack of selectivity towards different stereoisomers, we believe the following: (i) the intrinsic disorder of the isolated NLS peptides (derived from the cargos), as shown by NMR (Figure 1 and Figure 2), and (ii) the fact that the peptides remained disordered upon binding to the importin species (Figure 6) are the main reasons behind this lack of sensitivity towards stereoisomer recognition. Similar conclusions have been attained recently when the binding of polypeptide patches (the hot spots) of proteins towards their intact partners involved in different protein networks, but not in nuclear translocation, has been described [25]. The current view of protein–protein interactions is that progressively disordered complexes are expected to show decreased sensitivity to chirality [37]. The similarity of the binding affinities of L- and D-NLS peptides to importin observed in our case, as suggested by a comparison of BLI and ITC measurements for both sets of stereoisomers, suggests that importins were relatively insensitive to the stereochemistry or, more generally, to the degree of conformational flexibility of NLS regions. As the first example of a receptor insensitive to the chirality of a ligand in an intracellular pathway, it has been previously reported that a peptide corresponding to the NLS of c-Myc could function as an import signal both in L- and D-form [38]. In the same study, binding insensitive to L-/D-stereochemistry has been observed, even when the sequence of the peptide (A^321^AKRVKLD^328^, with numbering corresponding to wild-type c-Myc) has been synthesized in reverse order (i.e., DLKVRKAA). This indicates that Impα3, keeping in mind its intrinsic flexibility, is extraordinarily tolerant to disorder in the NLS and, since the plasticity in protein–protein interactions involving disordered regions (including indeed NLSs) could reveal the presence of strong evolutionary pressure [39], might also help to explain the binding versatility of importins from an evolutionary perspective.

## 4. Materials and Methods

### 4.1. Materials

Imidazole, Trizma base, DNase, SIGMAFAST protease tablets, NaCl, Ni^2+^-resin, 3-(trimethylsilyl) propionic acid-2,2,3,3-^2^H_4_-sodium salt (TSP) and ultra-pure dioxane were purchased from Sigma (Madrid, Spain). The β-mercaptoethanol was from BioRad (Madrid, Spain). Ampicillin and isopropyl-β-D-1-thiogalactopyranoside were obtained from Apollo Scientific (Stockport, UK). Triton X-100, dialysis tubing with a molecular weight cut-off of 3500 Da, and the SDS protein marker (PAGEmark Tricolor) were from VWR (Barcelona, Spain). Amicon centrifugal devices with a molecular weight cut-off of 3 or 30 kDa were from Millipore (Barcelona, Spain). The rest of the used materials were of analytical grade. Water was deionized and purified in a Millipore system.

### 4.2. Protein Expression and Purification

Impα3 and ∆Impα3 were purified as previously described [5,6,7,31,32]. Protein concentrations were determined by UV absorbance, by using an extinction coefficient at 280 nm estimated from the number of tyrosines and tryptophans in both importin species (6 tyrosine and 6 tryptophan residues) [40].

### 4.3. Peptide Design

The NLS regions for the protein sequences were predicted by using the web server cNLS Mapper [41,42], available at http://nls-mapper.iab.keio.ac.jp (accessed on 30 June 2023), as previously described [5,6,7,31,32]. The corresponding D-NLS peptides were synthesized as detailed hereafter, with the numbering corresponding to the position of the NLS in the intact protein.

(a) Two peptides were derived from the intrinsically disordered protein (IDP) NUPR1: YT^54^NRPSPGGHERKLVTKLQNSE^74^ (D-NLS-NUPR1) and its variant phosphorylated at Thr68 (D-NLS-NUPR1phospho). By designing these two peptides, we were able to address whether phosphorylation could alter the binding of the designed D-NLS, like their L-form counterparts [6]. The extra N-terminal tyrosine was added to the wild-type sequence because the presence of an aromatic residue aided in evaluating the peptide concentrations in absorbance measurements.

(b) A peptide was derived from the oligomeric NUPR1L, an orthologue of NUPR1, which is also an IDP: R^51^TRREQALRTNWPAPGGHERKVAQ^74^ (D-NLS-NUPR1L). By using this NLS, also coming from an IDP, we wanted to address whether the oligomeric nature of the parent protein [5] could alter the conformational and binding features of the isolated D-NLS region.

(c) A peptide was derived from the dimeric citrullinating enzyme PADI4 [43]: K^499^LFQEQQNEGHGEALLFEGIKKKKQQKI^526^ (D-NLS-PADI). By using this NLS, we wanted to address whether the folded nature of the parent protein could modulate the binding properties of isolated D-NLS (in the case of the L-form counterpart, the folded nature of the parent protein did not alter the binding properties of the isolated NLS [7]). Due to cost-effective reasons, this D-NLS peptide was minimally shorter than its L-form counterpart, namely Y^498^KLFQEQQNEGHGEALLFEGIKKKKQQKI^526^, which possessed an extra N-terminal tyrosine (at variance with the D-NLS peptides from NUPR1, the N-terminal Tyr498 is present in the wild-type sequence of PADI4). It is important to indicate that, in our previous study [7], we identified two possible NLS regions based on the PADI4 sequence; however, the first one, the peptide designed to correspond to the canonical NLS region (with the sequence P^56^PAKKKSTGSSTWPLDPGVEVTLTMKVASGS^86^), has a high tendency to aggregate, as we have previously shown by using DOSY-NMR experiments, 1D and 2D ^1^H-NMR and far-ultraviolet circular dichroism [7]; then, we decided not to synthesize the D-form counterpart of this peptide. Therefore, in this work, we are working with the peptide corresponding to the second predicted NLS region of PADI4, which was called NLS2-PADI4 in our previous study [7] and, in its D-form, is referred to as D-NLS-PADI.

The D-NLS peptides were produced by Genscript (Leiden, The Netherlands) with a purity higher than 95%. They were all acetylated and amidated at the N and C termini, respectively, to avoid fraying effects. Peptide concentrations were determined from the absorbance of tyrosine or tryptophan residues at 280 nm [40] and from that of phenylalanine at 260 nm [44].

### 4.4. Titration Fluorescence Experiments with the D-NLSs

Fluorescence spectra were collected on a Cary Varian spectrofluorometer (Agilent, Santa Clara, CA, USA), interfaced with a Peltier unit. All experiments were carried out at 25 °C. Following the standard protocols used in our laboratories, the samples were prepared the day before and left overnight at 5 °C; before experiments, samples were left for 1 h at 25 °C. A 1 cm pathlength quartz cell (Hellma, Kruibeke, Belgium) was used.

Protein or peptide samples were excited either at 280 or 295 nm. The slit widths for both excitation and emission were 5 nm, and experiments were carried out as described [45]. Appropriate blank corrections were made in all spectra.

For the titration between either Impα3 or ∆Impα3 and each D-NLS peptide, increasing amounts of the latter, in the concentration range 0–35 μM, were added to a solution with a fixed concentration of either Impα3/∆Impα3 (3 μM, in protomer units). Experiments were carried out at pH 8.0 (50 mM Tris buffer). In all cases, the appropriate blank corrections with the corresponding amounts of each peptide were subtracted. Spectra were corrected for inner-filter effects during fluorescence excitation [26]. Every titration was repeated twice. The variations in all cases were lower than 10%.

The samples were prepared the day before and left overnight at 5 °C; before the measurements, the samples were incubated for 1 h at 25 °C. The dissociation constant of the corresponding complex, *K*_d_, was calculated by fitting the binding isotherm constructed by plotting the observed fluorescence change as a function of peptide concentration to the general binding model, explicitly considering ligand depletion [27,28]:

(1) $$F=F_{0}+\frac{∆F_{max}}{2{\left[{Imp\alpha 3}_{species}\right]}_{T}}{{(\left[{Imp\alpha 3}_{species}\right]}_{T}+{\left[D-NLS\right]}_{T}+K}_{d})\;-\sqrt{\left({{{(\left[{Imp\alpha 3}_{species}\right]}_{T}+{\left[D-NLS\right]}_{T}+K}_{d})}^{2}-4{\left[{Imp\alpha 3}_{species}\right]}_{T}{\left[D-NLS\right]}_{T}\right)}$$

where *F* is the measured fluorescence at any particular concentration of the corresponding peptide after subtraction of the blank with the same concentration of D-NLS peptide; ∆*F*_max_ is the largest change in the fluorescence of the corresponding peptide when all polypeptide molecules were forming the complex, compared to the fluorescence of each isolated chain; *F*_0_ is the fluorescence intensity when no D-NLS was added; [*D-NLS*]_T_ is the total concentration of the corresponding peptide, which was varied during the titration; and [*Impα3_species_*]_T_ is the concentration of either Impα3 or ∆Impα3, which was kept constant during the titration. Fitting to the above equation was carried out by using KaleidaGraph (Synergy software).

### 4.5. Nuclear Magnetic Resonance (NMR) Spectroscopy

The NMR spectra were acquired at 10 °C on a Bruker Avance III spectrometer (Bruker GmbH, Mannheim, Germany), equipped with a triple-resonance probe and z-pulse field gradients. All NMR experiments with D-NLS peptides were carried out in 50 mM deuterated Tris buffer (not corrected for isotope effects), pH 7.2. Spectra were calibrated with TSP, by considering pH-dependent changes in its chemical shifts [46]; probe temperature was calibrated with pure methanol [46].

#### 4.5.1. 1D-^1^H-NMR Spectra

A total of 128 scans were acquired with 16 K acquisition points for the homonuclear 1D-^1^H-NMR spectra of each isolated D-NLS peptide at a concentration of ~1.2 mM. Water signal was suppressed with the WATERGATE sequence [47]. The spectra were processed after zero-filling and apodization with an exponential window.

#### 4.5.2. Translational Diffusion NMR (DOSY)

The typical D-NLS peptide concentrations in DOSY experiments were ~100 μM, and 128 scans were acquired, in which the gradient strength was varied linearly in sixteen steps, between 2 and 95% of the total power of the gradient coil. Measurements of the translational self-diffusion coefficient, *D*, were performed with the pulsed-gradient spin-echo sequence in the presence of 100% D_2_O. Details on the experimental conditions and fitting of the resulting curves have been described elsewhere [45]. Gradient strength was calibrated by using the value of *D* for the residual proton water signal in a sample containing 100% D_2_O, in a 5 mm tube [23]. Gradient length was 2.5 ms; the time between the two pulse gradients in the sequence was 250 ms; and the recovery delay between the bipolar gradients was 100 μs. The signals from methyl groups between 0.8 and 1.0 ppm were used for peak integration, for all the D-NLS peptides. A final concentration of 1% of dioxane, which was assumed to have a hydrodynamic radius *R*_h_ of 2.12 Å [23], was added to the solution of each peptide.

#### 4.5.3. 2D-^1^H-NMR Spectra

Two-dimensional spectra of D-NLS were acquired in phase-sensitive mode by using the time-proportional phase incrementation technique [48] and a spectral width of 6500 Hz; the concentration of the corresponding peptide was the same as used in the 1D-^1^H-NMR spectra. Standard TOCSY (by using a mixing time of 80 ms) [49] and NOESY experiments (by using a mixing time of 250 ms) [50] were acquired with a data matrix size of 2048 × 128 points. The decoupling in the presence of scalar interactions (DIPSI) spin-lock sequence [51] was used in the TOCSY experiments with a relaxation time of 1 s. A total of 96 scans were acquired per increment in the first dimension, and the water signal was removed by using the WATERGATE sequence [47]. NOESY spectra were collected with 128 scans per increment in the first dimension (and 2048 in the other ne), by using the WATERGATE sequence [47] and with a relaxation time of 1 s. Data for both experiments were zero-filled, resolution-enhanced with a square sine-bell window function optimized for each spectrum, and baseline-corrected. The ^1^H resonances were assigned by standard sequential assignment processes [20]. The chemical shift values of H_α_ protons in random-coil regions were obtained from tabulated data, corrected by neighboring residue effects for the L-amino acids [20,21,22].

### 4.6. Isothermal Titration Calorimetry (ITC)

Calorimetric titrations for testing the interaction of D-NLS peptides with either Impα3 or ΔImpα3 were carried out in an VP-ITC instrument (Microcal, Northhamptom, MA, USA). The importin species were loaded into the cell calorimeter and the corresponding D-NLS peptide in the syringe in Tris buffer 50 mM, pH 8.0. All experiments were carried out at 25 °C.

These twenty-nine ITC experiments involved sequential injections of microliter amounts (10 µL) of a stock solution of D-NLS peptide (in a concentration range of 150–200 µM) into the calorimetric cell (1.4 mL); the cell initially contained protein solution (15 µM for either Impα3 or ΔImpα3 in protomer units). To correct for the heat evolved due to the dilution effect for adding the ligand (peptide) solution to the calorimetric cell, an independent experiment was performed, in which the same peptide solution was injected into the calorimetric cell loaded with buffer. After correction for the dilution heat, the isotherm was fitted to a binding model that assumed the existence of a single set of binding sites, by using the software v1.41 provided by the manufacturer.

### 4.7. Biolayer Interferometry (BLI)

#### 4.7.1. Experimental Design

The association (*k*_on_) and dissociation (*k*_off_) rate constants of the binding of D-NLS peptides to either Impα3 or ∆Impα3 were determined by using a BLItz system (ForteBio, Pall, Barcelona, Spain) [52,53,54,55]. The buffer used in the experiments was that recommended by the manufacturer. Since Impα3 and ∆Impα3 had a His-tag, they were immobilized on the His-tag biosensors (Forte Bio) at a final concentration of 0.25 μM. The peptide concentrations were in a range from 1 to 10 μM during the association step. The general scheme of the protein association/dissociation reactions in the BLItz system for the D-NLS peptides was as follows: 30 s of initial baseline acquisition with the 10 × kinetics buffer (provided by the manufacturer); 120 s of loading either Impα3 and ∆Impα3 into the biosensor; 30 s of baseline acquisition with the 10 × kinetics buffer; 120 s of association of each peptide to the biosensor (which had been previously loaded with the importin species); and 120 s of monitoring of the dissociation of each peptide from the biosensor. All experiments were carried out at 25 °C.

#### 4.7.2. Fitting of the Sensorgrams

Fittings of the BLI sensorgrams was carried out by using KaleidaGraph, as previously described [56]. The interferometry response during the association step, *R(t)* (measured in response units, RU), and the binding rate, d*R(t)*/d*t*, could be used to evaluate the kinetics of the formation of the complex between Impα3/∆Impα3 and each D-NLS, according to:

(2) $$\frac{dR}{dt}=k_{on}\left[D-NLS\right]\left(R_{max}-R\left(t\right)\right)-k_{off}R(t)$$

where *R*_max_ is proportional to the total concentration of biosensor-bound importin species; and [*D-NLS*] represents the concentration of the corresponding D-NLS peptide.

In Equation (2), *R(t)* is given by:

(3) $$R\left(t\right)=\;R_{eq}-R_{eq}e^{\left(-k_{obs}\;\left(t-t_{0}\right)\right)}$$

where *R*_eq_ is the steady-state (or equilibrium) response obtained at infinite time, when d*R(t)*/d*t* = 0, and *t*_0_ = 180 s is the time at which the association step between biosensor-immobilized Impα3/∆Impα3 and D-NLS in the solution started. We fitted the experimentally obtained *R*(*t*) under any condition as:

(4) $$R\left(t\right)=\;R_{eq}-R_{eq}e^{\left(-k_{obs}\;\left(t-t_{0}\right)\right)}-{R’}_{eq}(t-t_{0})$$

where ${R^{\prime }}_{eq}$ was included to consider the lineal drift observed at longer times.

The *k*_obs_ showed a concentration-dependent kinetic rate, and it was used for the pseudo-first-order plots, where the value of *k*_obs_ was given by:

(5) $$k_{obs}=\;k_{on}\;\left[D-NLS\right]+k_{off}$$

The dissociation process was always fitted to a single exponential, with *R(t)* given by:

(6) $$R\left(t\right)=R_{1}\;e^{\left(-k_{off}\left(t-t_{0}\right)\right)}-{R^{\prime \prime }}_{eq}(t-t_{0})$$

where *t*_0_ = 300 is the time at which the dissociation of the D-NLS from the biosensor-bound Impα3/∆Impα3 started in our experimental set-up, *R*_1_ is the response level when dissociation starts, and the parameter ${R^{\prime \prime }}_{eq}$ considers the linear drift observed at longer times.

The experiments were repeated at least twice for each peptide with each importin species.

### 4.8. Molecular Docking

Molecular docking was used to model the binding of the D-NLS peptides to importin α3 by using ∆Impα3. The sole IBB-depleted species, ∆Impα3, was modeled on the basis of the crystallographic structure of the protein complexed with the NLS of the Ran-binding protein 3 (Protein Data Bank entry: 5XZX [57]), as previously described [7,31,58]. The peptides were built in elongated conformation by using USCF Chimera, version 1.12 [59], with the sole exceptions of Pro residues (dihedral angles ϕ = −60° and ϕ = 0°). The peptides were capped by acetylation and amidation at the N and C termini, respectively. The VMD software, version 1.9.3 [60], was used for displaying the protein–peptide complexes.

The simulations were performed by using the docking engine AutoDock Vina, version 1.2.5 [61]. The search volume was centered on the ∆Impα3 domain and had a grid spacing of 1 Å and size of 50 Å × 90 Å × 80 Å to encompass the whole protein surface. For restricting the very large number of degrees of freedom in the peptides to a maximum of 100 rotatable dihedral angles (about 3-times greater than the largest number recommended for the use of AutoDock Vina, i.e., 32 rotatable bonds [30]), a shorter version of D-NLS-PADI peptide was simulated (Q^502^EQQNEGHGEALLFEGIKKKKQ^523^) instead of the larger one used for the in vitro experiments (K^499^LFQEQQNEGHGEALLFEGIKKKKQQKI^526^). On the other hand, to improve the sampling, an exhaustiveness four-times larger than the default value of AutoDock Vina (i.e., 32 instead of 8) was used for all the peptides in the search algorithm. This linearly increases (by a factor of 4) the elapsed real time of the simulation but exponentially raises the probability of finding the global minimum in the energy landscape [62].

## 5. Conclusions

The stereochemistry of amino acids is fundamental for proteins, and L-chirality is predominant in the biological realm. However, nature also uses D-chirality in less common but biologically important polypeptides, including some short peptides or peptide-like regions. Peptides in D-form can also be exploited in bio-inspired materials, due to their unusual characteristics such as non-proteolytic and non-immunogenic properties, while still being able in many cases to exert convenient biological activity. In this work, we showed that isolated D-enantiomers of NLS of different proteins were capable of binding to the nuclear carrier importin α3, both in its wild-type form and without the IBB (which competes with NLS for the binding, by self-associating to the intact protein). By comparing the results with those previously obtained with the same NLS peptides in L-form and combining the results of different biophysical techniques (namely, fluorescence, ITC and BLI) in the L- and D- isomers, we found that chirality did not affect the disordered nature of the isolated NLS region, noticeably altering neither their binding location nor affinity. Taken together, these findings obtained by an array of orthogonal biophysical techniques suggest that importin α3 was remarkably tolerant to changes in both the stereochemistry and the degree of disorder of NLSs, still retaining their binding properties towards them.

The outcome of this work improves our understanding of the interaction of importin α3 with NLSs of cargo proteins and might offer new opportunities for pharmacological intervention by using D-NLS peptides to target importins, as a way to regulate nuclear translocation.

## Figures and Tables

**Table 1:** Hydrodynamic properties of the D-NLS peptides ^a^.

D-NLS Peptide ^b^	MW (Da)	*D* (cm^2^ s^−1^) × 10^6^ (*R*_h_, Å) ^c^	*D* (cm^2^ s^−1^) × 10^6^ (*R*_h_, Å) (L-NLS) ^d^	*R*_h_, Å ^e^
YT^54^NRPSPGGHERKLVTKLQNSE^74^ (D-NLS-NUPR1)	2511.78	1.78 ± 0.08 (9 ± 1)	1.85 ± 0.04 (11 ± 1)	13 ± 3
YT^54^NRPSPGGHERKLV**pT**KLQNSE^74^ (D-NLS-NUPR1phospho)	2541.78	2.2 ± 0.2 (10 ± 1)	1.89 ± 0.08 (11 ± 1)	13 ± 3
R^51^TRREQALRTNWPAPGGHERKVAQ^74^ (D-NLS-NUPR1L)	2815.15	2.0 ± 0.3 (10 ± 1)	1.74 ± 0.05 (12 ± 2)	14 ± 2
K^499^LFQEQQNEGHGEALLFEGIKKKKQQKI^526^ (D-NLS-PADI)	3297.80	1.0 ± 0.1 (13 ± 1)	0.96 ± 0.03 (15.1 ± 0.8)	15 ± 2

^a^ All the peptides were amidated at their C-termini, and acetylated at their N-termini. ^b^ The first peptide is the wild-type sequence of NUPR1, and the next peptide is a post-translational modification with a phospho-threonine at position 68 (indicated with a bold “pT”). ^c^ The R_h_ was determined by comparison with the translational diffusion coefficient of dioxane, *R*_h_, of 2.12 Å [23]. ^d^ Taken from the reported values in the literature for the L-NLS counterpart [5,6,7]. The value reported for the L-NLS-PADI counterpart is for the sequence Y^498^KLFQEQQNEGHGEALLFEGIKKKKQQKI^526^. The values within parentheses correspond to the *R*_h_ estimated from the *R*_h_ of the dioxane. ^e^ The hydrodynamic radius of the peptides, *R*_h_, were calculated for the from the experimentally-obtained scale law: *R*_h_ = (0.027 ± 0.01) MW^(0.50±0.01)^ [24], where MW is the molecular weight of the corresponding peptide used in this work, which is indicated in the second column, without including the acetyl nor amide moieties at the termini. The molecular weight of the phosphorylated peptide includes the phosphoric moiety at the threonine.

**Table 2:** Kinetic and thermodynamic parameters of the binding reaction of D-NLS peptides to Impα3 ^a^.

D-NLS Peptide	*k*_on_ (μM^−1^ s^−1^)	*k*_off_ (s^−1^)	*K*_d_ (μM) (=*k*_off_/*k*_on_) ^b^	*K*_d_ (μM) (ITC/Fluorescence)	*K*_d_ (μM) (ITC/Fluorescence) (L-NLS Peptide) ^c^
D-NLS-NUPR1	0.05 ± 0.01	- ^d^	- ^d^	- ^e^/- **^e^**	1.7
D-NLS-NUPR1phospho	0.010 ± 0.003	0.13 ± 0.02	13 ± 4	- ^e^/- **^e^**	27
D-NLS-NUPR1L	0.068 ± 0.007	0.069 ± 0.04	1.0 ± 0.6	2.7 ± 0.6/- **^e^**	12 ± 2/3 ± 1
D-NLS-PADI	0.045 ± 0.02	0.6 ± 0.1	15 ± 3	- ^e^/12 ± 5	23/4 ± 2

^a^ Errors in the rate constants are from fitting to Equation (5) (pseudo-first order plots). b Errors are calculated as propagation errors. c From the values reported in the indicated literature for the L-NLS counterpart [5,6,7]. The values of the Kd obtained by fluorescence (shown in bold) were obtained by fluorescence titrations, as those carried out in this work with the D-NLS peptides. d Not determined because the y-axis intercept of the pseudo-first order plot had a negative value. e In these cases for the corresponding peptides, no binding isotherm or fluorescence titration, which could be fitted by using Equation (1), could be detected (either by ITC or fluorescence, or by both techniques).

**Table 3:** Kinetic and thermodynamic parameters of the binding reaction of D-NLS peptides to ∆Impα3 ^a^.

D-NLS Peptide	*k*_on_ (μM^−1^ s^−1^)	*k*_off_ (s^−1^)	*K*_d_ (μM) (=*k*_off_/*k*_on_) ^b^	*K*_d_ (μM) (ITC/Fluorescence)	*K*_d_ (μM) (ITC/Fluorescence) (L-NLS Peptide) ^c^
D-NLS-NUPR1	0.11 ± 0.02	- ^d^	- ^d^	- ^e^/- **^e^**	0.95
D-NLS-NUPR1phospho	0.006 ± 0.001	0.05 ± 0.01	8 ± 2	- ^e^/- **^e^**	29
D-NLS-NUPR1L	0.0512 ± 0.0007	0.038 ± 0.05	0.7 ± 0.2	5 ± 1/- **^e^**	5.5 ± 0.9/5 ± 2
D-NLS-PADI	0.06 ± 0.01	0.37 ± 0.05	6 ± 1	- ^e^/16 ± 11	4.37/4 ± 1

^a^ Errors in the rate constants are from fitting to Equation (5) (pseudo-first order plots). ^b^ Errors are calculated as propagation errors. ^c^ From the values reported in the indicated literature for the L-NLS counterpart [5,6,7]. The values of the *K*_d_ obtained by fluorescence (shown in bold) were obtained by fluorescence titrations, as those carried out in this work with the D-NLS peptides. ^d^ Not determined because the y-axis intercept of the pseudo-first order plot had a negative value. ^e^ In these cases for the corresponding peptides, no binding isotherm or fluorescence titration curve, which could be fitted by using Equation (1), could be detected (either by ITC or fluorescence, or with both techniques).

**Table 4:** Binding affinities in the molecular docking to ∆Impα3 of the D-NLS peptides ^a^.

D-NLS Peptide ^b^	Length (Number of Residues)	Rotatable Bonds	Binding Affinity (kcal mol^−1^)
YT^54^NRPSPGGHERKLVTKLQNSE^74^ (D-NLS-NUPR1)	22	91	−5.2
YT^54^NRPSPGGHERKLV**pT**KLQNSE^74^ (D-NLS-NUPR1phospho)	22	92	−4.8
R^51^TRREQALRTNWPAPGGHERKVAQ^74^ (D-NLS-NUPR1L)	24	96	−5.3
Q^502^EQQNEGHGEALLFEGIKKKKQ^523^ (D-NLS-PADI)	22	100	−4.4

^a^ All the peptides were amidated at their C-termini, and acetylated at their N-termini. ^b^ The sequence of peptide D-NLS-PADI used in simulation is shorter than the one used in the experiments in vitro, as the latter is K^499^LFQEQQNEGHGEALLFEGIKKKKQQKI^526^.

## Data Availability

The data are available from the corresponding authors upon request.

## Supplementary Materials

The following supporting information can be downloaded at: https://www.mdpi.com/article/10.3390/ijms26167818/s1.

## Abbreviations

The following abbreviations are used in this manuscript:

ARM	Armadillo
BLI	Biolayer interferometry
DIPSI	Decoupling in the presence of scalar interactions
DOSY	Diffusion-ordered spectroscopy
IBB	Importin β-binding domain
Impα3	Human importin α3 isoform (residues 1–521)
∆Impα3	IBB-depleted species of Impα3 (residues 64–521 of the intact protein)
ITC	Isothermal titration calorimetry
NLS	Nuclear localization signal
D-NLS	D-enantiomeric form of isolated NLS region
D-NLS-NUPR1	D-enantiomeric, isolated NLS region of NUPR1 (residues 54–74 of the intact protein)
D-NLS-NUPR1phospho	D-enantiomeric, isolated NLS region of NUPR1 with phosphorylated Thr68
D-NLS-NUPR1L	D-enantiomeric, isolated NLS region of NUPR1L (residues 51–74 of the intact protein)
D-NLS-PADI	D-enantiomeric, isolated, non-canonical NLS of PADI4 (residues 498–526 of the intact protein)
NMR	Nuclear magnetic resonance
NOESY	Nuclear Overhauser effect spectroscopy
NPC	Nuclear pore complex
NUPR1	Nuclear protein 1
NUPR1L	Paralogue of NUPR1
PADI	Protein arginine deiminase
TOCSY	Total correlation spectroscopy
TSP	3-(trimethylsilyl) propionic acid-2,2,3,3-^2^H_4_-sodium salt
