# Supplementary material for: Importin α3 Is Tolerant to Nuclear Localization Signal Chirality
Source: Int J Mol Sci. 2025 Aug 13;26(16):7818. doi: 10.3390/ijms26167818 (PMC12386367; doi:10.3390/ijms26167818)
Supplement: Supplementary file 1 [file ijms-26-07818-s001.zip › ijms-3769299-supplementary.pdf]

Supplementary Material

Importin α3 is tolerant to nuclear localization signal chirality

Felipe Hornos, Bruno Rizzuti and José L Neira

FIGURE S1: **Binding of the D-NLS peptides to ΔImpα3 monitored by fluorescence.** Fluorescence titrations of ΔImpα3 with (A) D-NLS-NUPR1phospho; (B) D-NLS-NUPR1; (C) D-NLS-NUPR1L; and (D) D-NLS-PADI. The fluorescence intensity on the y-axis is the relative fluorescence intensity after removal of the corresponding blank. Experiments were carried out at 25 ºC.

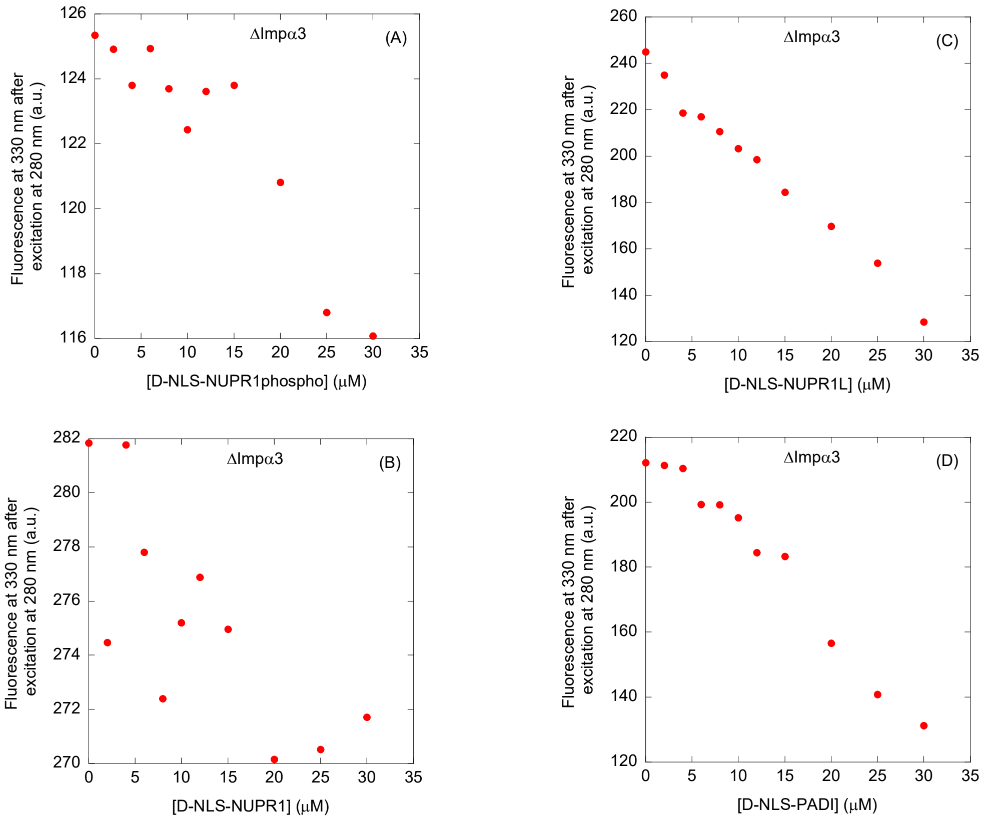

FIGURE S2: **Binding of the D-NLS peptides to ΔImpα3 monitored by ITC.** Binding isotherms of ΔImpα3 with (A) D-NLS-NUPR1L; (B) D-NLS-NUPR1; (C) D-NLS-NUPR1phospho; and (D) D-NLS-PADI. Continuous line corresponds to the fitting curve according to a single ligand binding site interaction model. Experiments were carried out at 25 ºC.

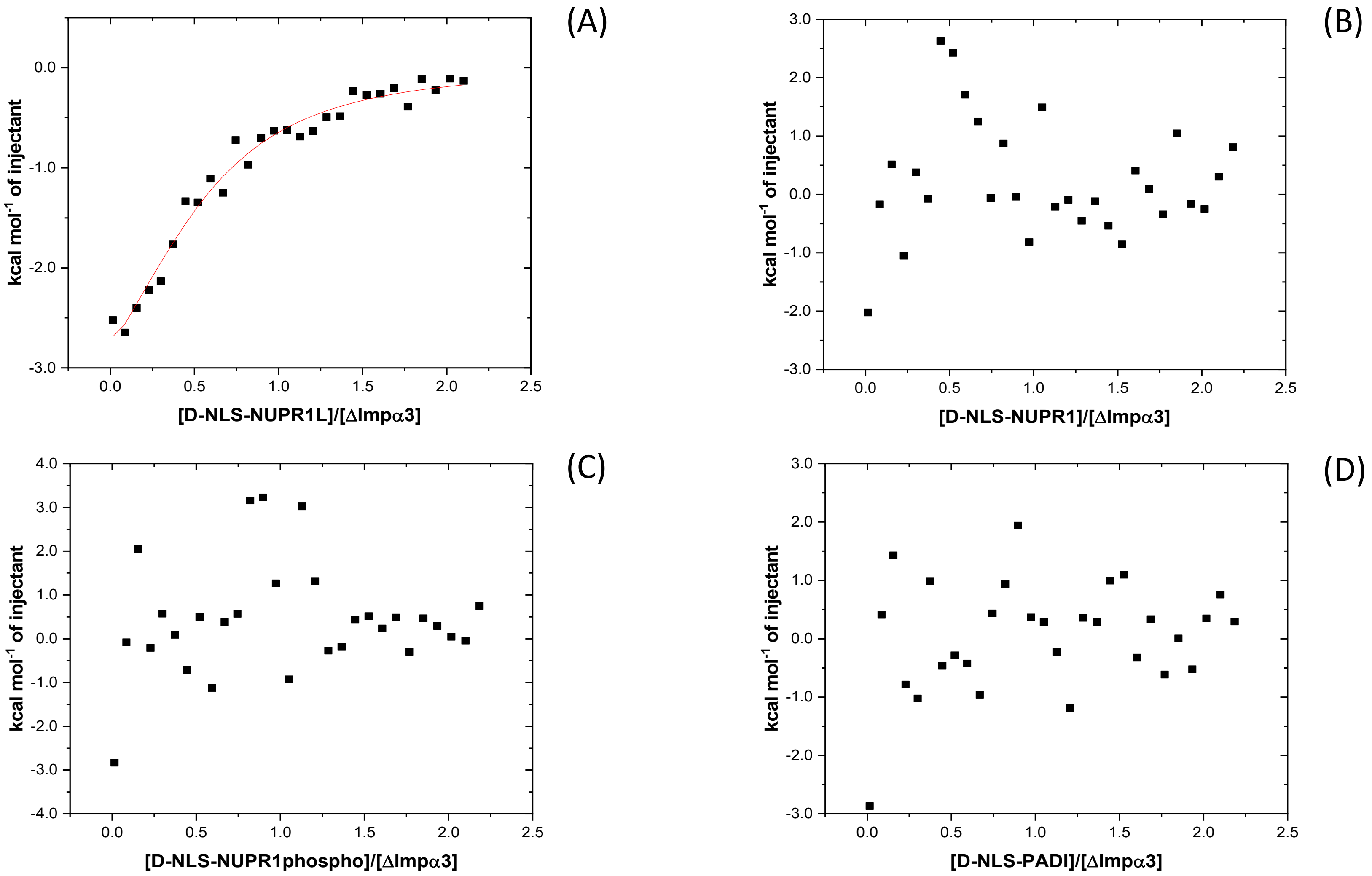

FIGURE S3: **Binding of selected D-NLS peptides to importin species as measured by BLI**: Binding step of the sensorgrams for different peptide concentrations of the binding of (A) Impα3 to D-NLS-NUPR1; and (B) ΔImpα3 to D-NLS-PADI. Experiments were carried out at 25 ºC.

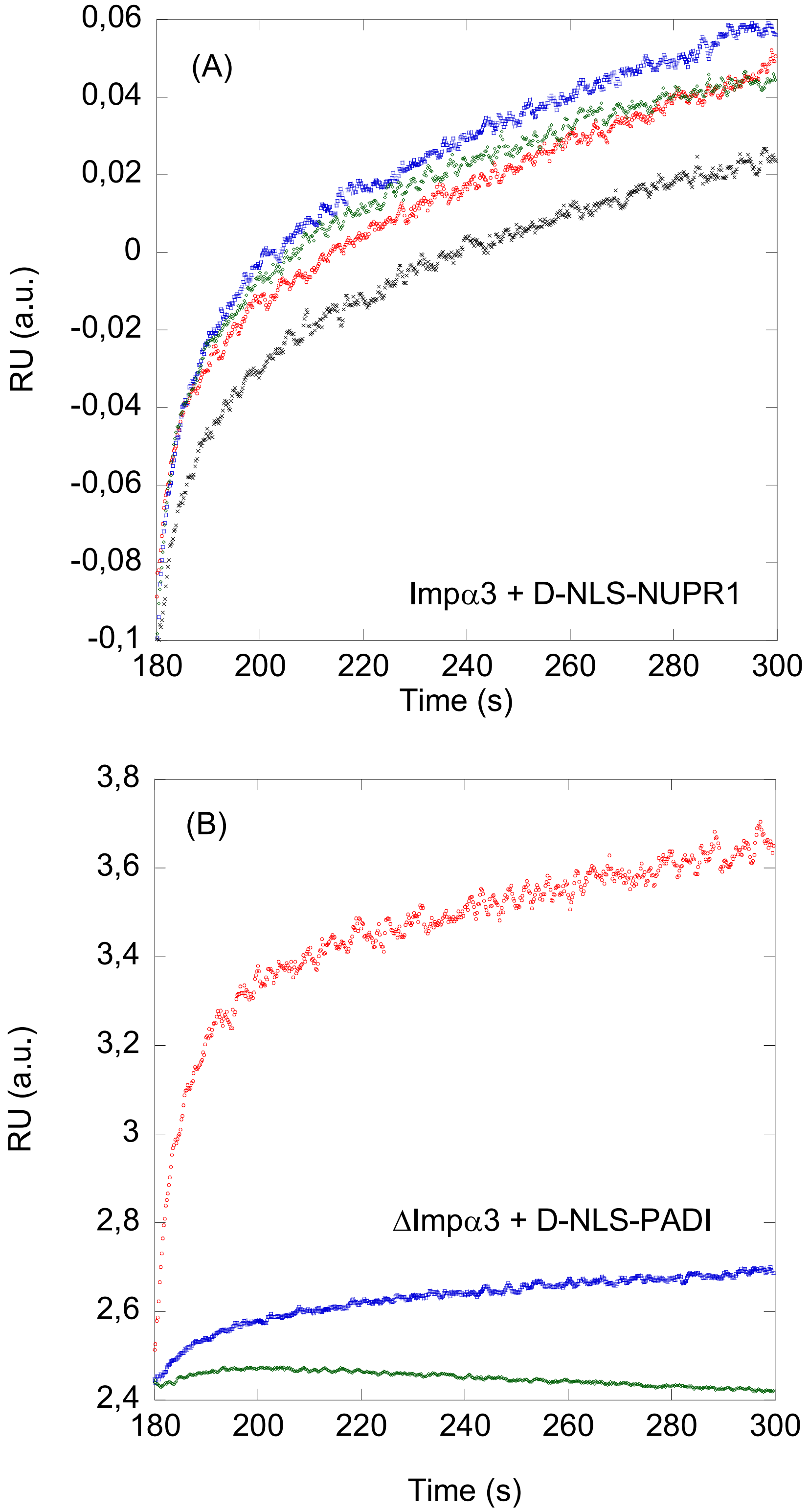

SUPPPORTING TABLES

Table S1: Chemical shifts (δ, ppm from TSP) of D-NLS-NUPR1L in aqueous solution (pH 7.0, 10 ºC)[a]

	NH	Hα	Hβ2	Hβ3	Hγ2	Hγ3	Hδ2	Hδ3	Hε	Hζ
Ac-Arg51	8.28	4.32 (-0.09)	1.82		1.66					
Thr52	8.19	4.23 (-0.11)	4.13		1.16 (Me)					
Arg53	8.54*	4.29*								
Arg54	8.54*	4.30*								
Glu55	8.40	4.35 (0.09)	2.02		2.36					
Gln56	8.18*	4.32*	2.20*							
Ala57	8.31	4.28 (-0.01)	1.37 (Me)							
Leu58	8.18*	4.32*	1.84		1.64		0.84 (Me)			
Arg59	8.56	4.33 (-0.11)	1.83		1.59		3.36			
Thr60	8.30	4.34 (0.05)	4.26		1.22 (Me)					
Asn61	8.47	4.73 (0.08)	2.52; 2.68							
Trp62 (t)	8.23	4.72 (-0.25)	3.06; 3.31						10.04 (NH); 7.23 (2H); 7.59 (7H); 7.17 (5H); 7.20 (6H) 7.69 (4H)	
Trp62 (c)									10.22 (NH); 7.22 (2H); 7.57 (7H)	
Pro63 (t)		4.55 (0.26)	1.95		2.23		3.52; 3.78			
Pro63 (c)			2.05		2.28		3.71; 3.86			
Ala64 (t)	8.30	4.53 (0.11)	1.36 (Me)							

Ala64 (c)						
Pro65		4.49 (0.02)	1.99	2.25	3.64; 3.82	
Gly66	8.63	3.96 (0.01)				
Gly67	8.38	3.96 (-0.01)	1.78	1.45	0.87 (Me)	
His68	8.00	4.59 (-0.06)	3.13		7.30 (C4H)	8.63 (C2H)
Glu69*	8.57*	4.34*	2.02; 2.36	2.64		
Arg70*	8.55*	4.24*				
Lys71	8.56	4.30 (-0.04)	1.77	1.61		
Val72	8.32	4.10 (0.03)	2.03	0.90 (Me)		
Ala73	8.57	4.29 (-0.02)	1.39 (Me)			
Gln74-Am	8.59	4.27 (-0.02)	1.98; 2.38	2.68; 2.85		

[a] The (*) indicates those residues whose resonances could not be unambiguously assigned. The (c) and (t) indicate the cis and trans conformations, respectively.The N terminus was acetylated and the C terminus was amidated. For the H_α proton column, the values within parenthesis are the conformational shifts (δ_{res}-δ_{rc}), only shown for those protons unambiguously assigned. The random-coil values for the sequence were obtained from the L-peptides: https://spin.niddk.nih.gov/bax/nmrserver/Poulsen_rc_CS/).

Table S2: Chemical shifts (δ, ppm from TSP) of wild-type D-NLS-NUPR1 in aqueous solution (pH 7.0, 10 ºC)[a]

	NH	H_{α}	$H_{\beta2}$	$H_{\beta3}$	$H_{\gamma2}$	$H_{\gamma3}$	$H_{\delta2}$	$H_{\delta3}$	H_{ε}	H_{ζ}
Ac-Tyr	8.41	4.56 (-0.14)	2.97				7.16		6.86	
Thr54	8.31	4.14 (-0.17)	4.10		0.84 (Me)					
Asn55	8.51	4.72 (0.05)	2.77				6.98; 7.60			
Arg56	8.29	4.67 (0.05)	1.89		1.76					
Pro57		4.38 (-0.13)	1.97		2.27		3.62; 3.80			
Ser58 (t)	8.72	4.72 (0.00)	3.96							
Ser58 (c)	8.65	4.69 (-0.03)	3.84							
Pro59		4.46 (-0.06)	1.97		2.25		3.73; 3.83			
Gly60	8.59	3.95 (0.00)								
Gly61	8.38	3.87 (-0.10)								
His62	8.55	4.72 (0.07)	3.14; 3.24				7.28 (C4H)		8.56 (C2H)	
Glu63	8.72	4.30 (0.03)	1.97		2.27					
Arg64	8.57	4.26 (-0.03)	1.61; 1.75							
Lys65	8.52	4.24 (-0.06)	1.96		1.62					
Leu66	8.51	4.42 (0.04)	1.56				0.87 (Me)			
Val67	8.23	4.26 (0.09)	2.09		0.95 (Me)					
Thr68	8.37	4.30 (-0.05)	4.21		1.21 (Me)					

Lys69	8.51	4.38 (0.05)	1.71	1.65	1.38	
Leu70	8.42	4.32 (-0.01)	1.86	1.63	0.92 (Me)	
Gln71	8.57	4.34 (-0.01)	1.98	2.26; 2.58		6.95; 7.60
Asn72	8.61	4.71(-0.05)	2.84		7.02; 7.71	
Ser73	8.46	4.42 (-0.04)	3.89			
Glu74	8.60	4.30 (0.00)	2.01; 2.10	2.30		

[a] The (c) and (t) indicate the cis and trans conformations.The N terminus was acetylated and the C terminus was amidated. For the H_α proton column, the values within parenthesis are the conformational shifts (δ_{res}-δ_{rc}), only shown for those protons unambiguously assigned. The random-coil values for the sequence were obtained from the L-peptides: https://spin.niddk.nih.gov/bax/nmrserver/Poulsen_rc_CS/).

Table S3: Chemical shifts (δ, ppm from TSP) of wild-type D-NLS-NUPR1phospho in aqueous solution (pH 7.0, 10 ºC)[a]

	NH	H_α	$H_{\beta 2}$	$H_{\beta 3}$	$H_{\gamma 2}$	$H_{\gamma 3}$	$H_{\delta 2}$	$H_{\delta 3}$	H_ε	H_ζ
Ac-Tyr	8.41	4.55 (-0.15)	2.97				7.15		6.86	
Thr54	8.31	4.14 (-0.17)	4.09		0.89 (Me)					
Asn55	8.51	4.67 (-0.02)	2.74				6.97; 7.62			
Arg56	8.30	4.63 (0.01)	1.89		1.72					
Pro57		4.38 (-0.13)	1.91		2.29		3.62; 3.80			
Ser58 (t)	8.71	4.71 (-0.01)	3.96							
Ser58 (c)	8.65	4.70 (-0.02)	3.83							
Pro59		4.41 (-0.11)	1.91		2.29		3.73; 3.83			
Gly60	8.58	3.96 (0.01)								
Gly61	8.36	3.91 (-0.06)								
His62	8.55	4.72 (0.07)	3.15; 3.28				7.19 (C4H)		8.63 (C2H)	
Glu63	8.72	4.32 (0.05)	1.95;1.98		2.27					
Arg64	8.57	4.30 (0.01)	1.61; 1.75							
Lys65	8.51	4.27 (-0.03)	1.96		1.62		1.36			
Leu66	8.49	4.32 (-0.06)	1.61; 1.75				0.89 (Me)			
Val67	8.34	4.17 (0.0)	2.00		0.96 (Me)					
**pThr68**	8.62	4.39 (0.32)	4.57		1.28 (Me)					

Lys69	8.36	4.38 (0.05)	1.78	1.70	1.43	
Leu70	8.31	4.34 (-0.03)	1.72	1.70	0.86 (Me)	
Gln71	8.57	4.32 (-0.03)	2.00	2.32		6.95; 7.60
Asn72	8.60	4.72 (-0.04)	2.82		7.01; 7.72	
Ser73	8.47	4.42 (-0.03)	3.88; 3.95			
Glu74	8.60	4.30 (0.00)	2.07; 2.14	2.35		

[a] The phospphrylated threonine iss indicated in bold. The (c) and (t) indicate the cis and trans conformations.The N terminus was acetylated and the C terminus was amidated. For the H_{α} proton column, the values within parenthesis are the conformational shifts (δ_{res}-δ_{rc}), only shown for those protons unambiguously assigned. The random-coil values for the sequence were obtained from the L-peptides: https://spin.niddk.nih.gov/bax/nmrserver/Poulsen_rc_CS/).

Table S4: Chemical shifts (δ, ppm from TSP) of wild-type D-NLS-PADI in aqueous solution (pH 7.0, 10 ºC)[a]

	NH	H_{α}	$H_{\beta 2}$	$H_{\beta 3}$	$H_{\gamma 2}$	$H_{\gamma 3}$	$H_{\delta 2}$	$H_{\delta 3}$	H_{ε}	H_{ζ}
Ac-Ly499	8.41	4.69 (0.49)	1.80				7.15		6.86	
Leu500	8.10	4.27 (0.05)	1.53; 1.61		1.43		0.83 (Me)			
Phe501	8.31	4.55 (-0.11)	3.06				7.24		7.35	7.26
Gln502	8.56*	4.24*								
Glu503	8.58*	4.24*								
Gln504	8.51*	4.24*								
Gln505	8.51*	4.24*								
Asn506	8.56	4.68 (-0.02)	2.80							
Glu507	8.41	4.24 (-0.03)								
Gly508	8.46	3.91 (-0.04)								
His509	8.51	4.75 (0.06)	3.18; 3.36				7.32 (C4H)		8.63 (C2H)	
Gly510	8.66	3.93 (-0.06)								
Glu511	8.62	4.29 (0.05)								
Ala512	8.45	4.22 (-0.07)	1.41 (Me)							
Leu513	8.20	4.27 (0.01)	1.63		1.41		0.88 (Me)			
Leu514	8.28	4.27 (-0.03)	1.61		1.44		0.87 (Me)			
Phe515	8.28	4.61 (-0.07)	3.06				7.24		7.35	7.26

Glu516	8.34*	4.24*	2.01*	2.34*	
Gly517	8.00	3.88 (-0.06)			
Ile518	8.00	4.28 (0.15)	1.94	1.24; 1.56	0.87 (Me)
Lys519	8.40*	4.27*	1.75*		7.01; 7.72
Lys520	8.34*	4.27*	1.75*		
Lys521	8.34*	4.27*	1.75*		
Lys522	8.34*	4.27*	1.75*		
Gln523	8.51*	4.24*			
Gln524	8.51*	4.24*			
Lys525	8.34*	4.27*	1.75*		
Ile526	8.40	4.26 (0.12)	1.74	1.34	0.91 (Me)

[a] The phospphrylated threonine iss indicated in bold. The (c) and (t) indicate the cis and trans conformations.The N terminus was acetylated and the C terminus was amidated. For the H_{α} proton column, the values within parenthesis are the conformational shifts (δ_{res}-δ_{rc}), only shown for those protons unambiguously assigned. The random-coil values for the sequence were obtained from the L-peptides: https://spin.niddk.nih.gov/bax/nmrserver/Poulsen_rc_CS/).
